# Levetiracetam Ameliorates Doxorubicin-Induced Chemobrain by Enhancing Cholinergic Transmission and Reducing Neuroinflammation Using an Experimental Rat Model and Molecular Docking Study

**DOI:** 10.3390/molecules27217364

**Published:** 2022-10-29

**Authors:** Vasudevan Mani, Minhajul Arfeen, Syed Imam Rabbani, Ali Shariq, Palanisamy Amirthalingam

**Affiliations:** 1 Department of Pharmacology and Toxicology, College of Pharmacy, Qassim University, Buraydah 51452, Saudi Arabia; 2 Department of Medicinal Chemistry and Pharmacognosy, College of Pharmacy, Qassim University, Buraydah 51452, Saudi Arabia; 3 Department of Pathology, College of Medicine, Qassim University, Buraydah 51452, Saudi Arabia; 4 Department of Pharmacy Practice, Faculty of Pharmacy, University of Tabuk, Tabuk 47512, Saudi Arabia

**Keywords:** cognitive impairment, doxorubicin, levetiracetam, spatial memory, neuroinflammation, molecular docking

## Abstract

Cancer chemotherapy-induced cognitive impairment (chemobrain) is a major complication that affects the prognosis of therapy. Our study evaluates the nootropic-like activity of levetiracetam (LEVE) against doxorubicin (DOX)-induced memory defects using in vivo and molecular modelling. Rats were treated with LEVE (100 and 200 mg/kg, 30 days) and chemobrain was induced by four doses of DOX (2 mg/kg, i.p.). Spatial memory parameters were evaluated using an elevated plus maze (EPM) and Y-maze. Additionally, acetylcholinesterase (AChE) and the neuroinflammatory biomarkers cyclooxygenase-2 (COX-2), prostaglandin E2 (PGE2), nuclear factor-κB (NF-κB), and tumor necrosis factor-alpha (TNF-α) were analyzed using brain homogenate. PharmMapper was used for inverse docking and AutoDock Vina was used for molecular docking. LEVE treatment significantly diminished the DOX-induced memory impairment parameters in both the EPM and Y-maze. In addition, the drug treatment significantly reduced AChE, COX-2, PGE2, NF-κB, and TNF-α levels compared to DOX-treated animals. The inverse docking procedures resulted in the identification of AChE as the potential target. Further molecular modelling studies displayed interactions with residues Gly118, Gly119, and Ser200, critical for the hydrolysis of ACh. Analysis of the results suggested that administration of LEVE improved memory-related parameters in DOX-induced animals. The ‘nootropic-like’ activity could be related to diminished AChE and neuroinflammatory mediator levels.

## 1. Introduction

Cancer is considered the second leading cause of death worldwide. Several causes have been linked to cancer, such as tobacco and alcohol consumption, unhealthy lifestyle and diet, viral and bacterial infection, exposure to pollution, irradiation, and chemicals. Due to changes in the world’s population demographic and environmental dynamics, cancer could cause around 21.4 million deaths by 2030 [1]. The mortality due to cancer in Saudi Arabia is also experiencing an upward trend. A study conducted in 2020 indicated 10,518 deaths due to cancer, with 24,485 new cases recorded in that year [2]. The common types of cancer reported in the population are breast, colon, and prostate. The risk factors associated with these are: genetic, environmental, age, gender, and inflammatory disorders [3].

Doxorubicin (DOX) is one of the important anticancer drugs used in treating several types of cancers, such as breast, lung, stomach, liver, endometrium, and bladder [4]. The drug is reported to produce its cytotoxic effect through multiple pathways, and important among them are inhibition of topoisomerase-II, and intercalation of DNA leading to a blockade in the synthesis of nuclear components. These actions are mediated due to the production of a variety of oxygen-free radicals [5]. Similarly to others, DOX-related chemotherapy is associated with several complications. One of these is neurological-related problems; doxorubicin can cause severe neurotoxicity by stimulating the generation of inflammatory and pro-inflammatory mediators in the periphery, even if it only passes through the blood-brain barrier (BBB) in a limited way [6]. DNA damage, oxidative stress, apoptotic dysregulation, modulation in neurotransmitter levels, mitochondrial dysfunction, glial cell connections, neurogenesis suppression, and epigenetic variables are among the other pathways linked to neuronal impairments [7]. Neurological dysfunction in patients is manifested as problems with thinking, recalling events, and performing tasks; these are collectively referred to as chemobrain. They are considered important limitations of DOX chemotherapy that have been reported to affect the treatment outcome [8].

Attempts in the past have been made to minimize the complications of DOX chemotherapy [8,9]. These studies have suggested that combining drugs with anti-inflammatory, nootropic, neuroprotective and anti-apoptotic actions could minimize the incidences of chemobrain [10]. Levetiracetam (LEVE) is an (S)-enantiomer of piracetam, which is the first approved drug for nootropic effect. However, LEVE is licensed for treating epilepsy in patients sixteen years of age and above. LEVE was reported to possess a novel mechanism of action in epilepsy by binding to synaptic proteins (SV2A) and modulating the synaptic vesicle causing exocytosis and neurotransmitter release [11]. Earlier studies have indicated that the administration of LEVE can relieve migraine, neuropathic pain, bipolar disorders, and anxiety [12]. 

Preclinical studies have indicated that long-term treatment of LEVE can improve cognitive abilities such as visual short-term memory, working memory, motor functions, psychomotor speed, and concentration [13]. The possible mechanism suggested as LEVE administration prolonged the reduction of abnormal spike activity that was found to ameliorate impairment in learning and memory and fully reversed deficits in synaptic transmission as well as spasticity in the hippocampus [14]. One of the mechanisms for this action is suggested to be the reduction in the neuroinflammatory pathways in the brain’s cells [15]. Based on this information, the present study was designed to evaluate the nootropic-like activity of LEVE in DOX-induced memory defects and determine the possible mechanism by estimating the biomarker level in experimental animals using the molecular modelling approach.

## 2. Results

### 2.1. Effect of Levetiracetam on DOX-Induced Cognitive Impairment Parameters Using an Elevated Plus-Maze (EPM) Test

**Figure 1:**
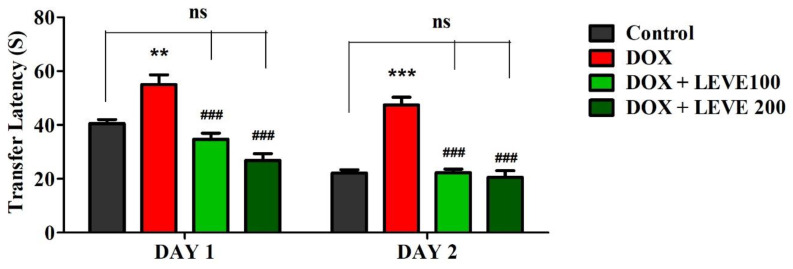
Effect of levetiracetam (LEVE) on day-1 and day-2 transfer latency of doxorubicin (DOX)-induced rats using elevated plus-maze (EPM). The results are expressed by mean ± SEM (n = 7). One-way ANOVA [*F*(3,24) = 21.27, *p* < 0.001 for day-1 and *F*(3,24) = 37.72, *p* < 0.001 for day-2 of EPM test] followed by Tukey–Kramer multiple comparisons test. ** *p* < 0.01, and *** *p* < 0.001 as compared to the control group; ns—not significant as compared to the control group; ^###^ *p* < 0.001 as compared to the DOX-induced group.

The observations recorded on day 1 and day 2 using the EPM are summarized in Figure 1. An analysis of day-1 results indicated that treatment with DOX (2 mg/kg; i.p.) significantly (*p* < 0.01) increased the transfer latency (TL) compared to the control animals. LEVE at both tested doses (100 and 200 mg/kg; p.o.) significantly (*p* < 0.001) minimized the DOX-elevated TL duration associated with DOX-induced animals. The day-2 analysis indicated that DOX-induced animals showed a significant (*p* < 0.001) increase in the TL duration compared to saline-treated control animals. The administration of LEVE at both doses (100 and 200 mg/kg; p.o.) significantly (*p* < 0.001) suppressed the TL duration compared to DOX-treated rats.

### 2.2. Effect of Levetiracetam on DOX-Induced Cognitive Impairment Parameters Using Y-Maze Test

**Figure 2:**
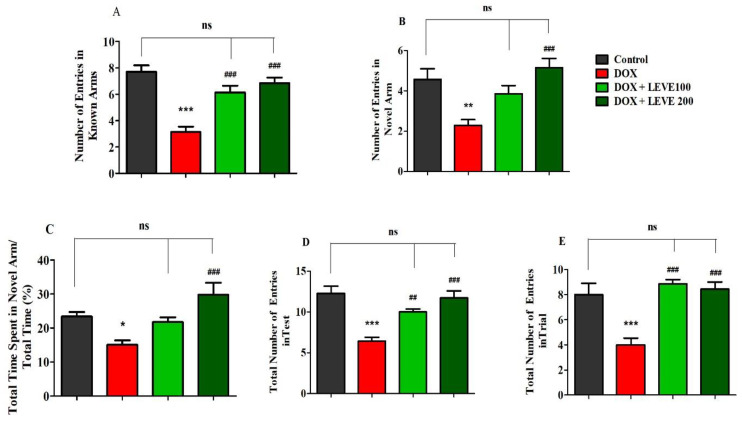
Effect of levetiracetam (LEVE) on (**A**) the number of entries in known arms in test, (**B**) the number of entries in novel arm in test, (**C**) the percentage of time spent in the novel arm in test, (**D**) the total number of entries in the test, and (**E**) the total number of entries in the trial of a doxorubicin (DOX)-induced rat model using Y-maze. The results are expressed by mean ± SEM (n = 7). One-way ANOVA [*F*(3,24) = 19.52, *p* < 0.001 for the number of entries in the known arm; *F*(3,24) = 8.324, *p* < 0.001 for the number of entries in the novel arm; *F*(3,24) = 8.176, *p* < 0.001 for the percentage of time spend in the novel arm; *F*(3,24) = 14.16, *p* < 0.001 for the total number of entries in the test; *F*(3,24) = 13.08, *p* < 0.001 for the total number of entries in the trial] followed by Tukey–Kramer multiple comparisons test. * *p* < 0.05, ** *p* < 0.01 and *** *p* < 0.001 as compared to the control group; ns—not significant as compared to the control group; ^##^ *p* < 0.01 and ^###^ *p* < 0.001 as compared to the DOX-induced group.

Figure 2 represents the data recorded for LEVE (100 and 200 mg/kg, p.o.) against DOX-induced animals using Y-maze. The observations suggested that DOX (2 mg/kg)-induced rats showed a significant (*p* < 0.001) reduction in the number of entries in the known arm compared to control animals. LEVE at both doses (100 and 200 mg/kg, p.o.) exhibited significant improvement (*p* < 0.001) in the number of entries in the known arm compared to DOX-treated rats (Figure 2A). In the novel arm, DOX-induced rats again showed a significantly decreased number of entries (*p* < 0.001) compared to the control group. Besides, the administration of LEVE at a higher dose (200 mg/kg, p.o.) enhanced the number of entries significantly (*p* < 0.001) compared to the DOX-induced group (Figure 2B). The data on the total time spent in the novel arm indicated that the administration of DOX reduced the total time significantly (*p* < 0.05) compared to the control animals. Further, when LEVE was tested in two doses, only the higher dose (200 mg/kg, p.o.) increased the total time spent significantly (*p* < 0.001) compared to the DOX-induced group (Figure 2C). The data on the total number of entries during the test session indicated that DOX-treated animals had significantly (*p* < 0.001) declined entry numbers compared to the control. LEVE treatment significantly elevated the number of entries at the lower dose (200 mg/kg, p.o.; *p* < 0.01) as well as at the higher dose (200 mg/kg, p.o.; *p* < 0.001) compared to the DOX-induced group (Figure 2D). The total number of entries during the trial session is represented in Figure 2E and the data suggested that DOX-induced rats have a significantly (*p* < 0.001) lower number compared to the control. On the other hand, LEVE at both doses (100 and 200 mg/kg, p.o.) significantly improved (*p* < 0.001) the number of total entries compared to data obtained for the DOX-induced animals.

### 2.3. Effect of Levetiracetam on Acetylcholinesterase (AChE) Levels in the Brain Homogenate of DOX-Treated Animals

**Figure 3:**
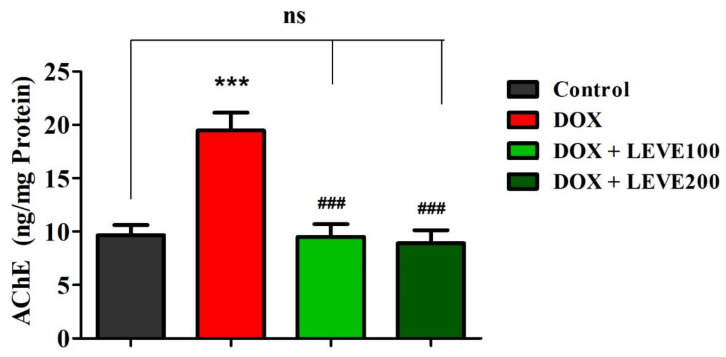
Effect of levetiracetam (LEVE) on acetylcholinesterase (AChE) levels in brain homogenates of the doxorubicin (DOX)-induced rat model. The results are expressed by mean ± SEM (n = 7). One-way ANOVA [*F*(3,24) = 15.44, *p* < 0.001] followed by Tukey–Kramer multiple comparisons test. *** *p* < 0.001 as compared to the control group; ns—not significant as compared to the control group; ^###^ *p* < 0.001 as compared to the DOX-induced group.

The effect of LEVE on the AChE levels in the DOX-induced animals is represented in Figure 3. A comparison of the data indicated that intraperitoneal injections of DOX at 2 mg/kg significantly (*p* < 0.001) elevated the AChE levels in the brain homogenate of animals in association with the control animals. LEVE at both the tested doses (100 and 200 mg/kg, p.o.) significantly (*p* < 0.001) diminished the AChE levels compared to the DOX-induced group in the brain homogenate of animals.

### 2.4. Effect of Levetiracetam on Neuroinflammatory Biomarker Levels in the Brain Homogenate of DOX-Treated Animals

**Figure 4:**
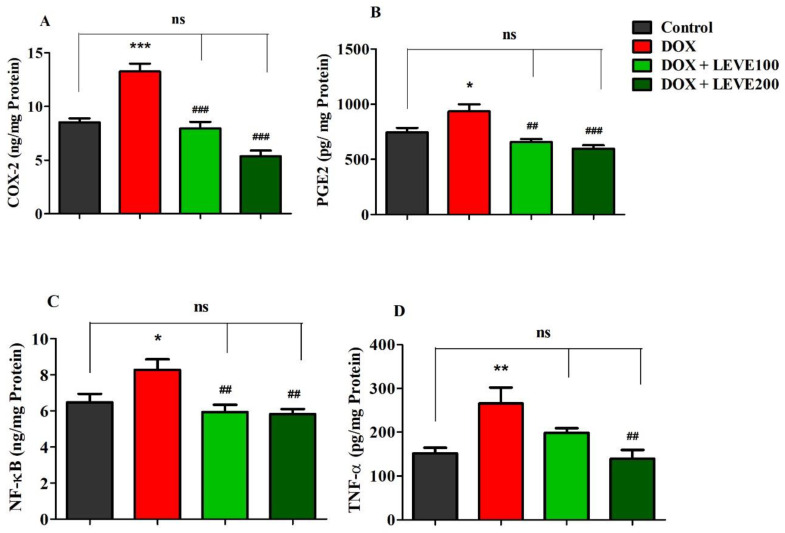
Effect of levetiracetam (LEVE) on inflammatory parameters, such as (**A**) COX-2, (**B**) PGE2, (**C**) NF-κB, and (**D**) TNF-α, in a doxorubicin (DOX)-induced rat model. The results are expressed by mean ± SEM (n = 7). One-way ANOVA [*F*(3,24) = 32.82, *p* < 0.001 for COX-2; *F*(3,24) = 10.81, *p* < 0.001 for PGE2; *F*(3,24) = 6.421, *p* < 0.01 for NF-κB; *F*(3,24) = 6.555, *p* < 0.01 for TNF-α] followed by Tukey–Kramer multiple comparisons test. * *p* < 0.05, ** *p* < 0.01, and *** *p* < 0.001 as compared to the control group; ns—not significant as compared to the control group; ^##^ *p* < 0.01 and ^###^ *p* < 0.001 as compared to the DOX-induced group.

Targeted neuroinflammatory parameters including COX-2, PGE2, NF-κB, and TNF-α were estimated in DOX-induced rat’s brains (Figure 4). An analysis of cyclooxygenase-2 (COX-2) levels in brain homogenate indicated that DOX treatment significantly (*p* < 0.001) increased when a comparison was performed with the control group. Administration of both doses of LEVE (100 and 200 mg/kg, p.o.) significantly reduced the levels of COX-2 compared to DOX-induced animals (Figure 4A). A comparison of brain homogenate levels of prostaglandin E2 (PGE2) between the control and DOX-induced animals revealed that four-weekly doses of DOX significantly (*p* < 0.05) elevated the enzyme levels. LEVE at the lower dose (100 mg/kg, p.o.) reduced the levels of PGE2 significantly (*p* < 0.01) and the higher dose (200 mg/kg, p.o.) showed further reduction (*p* < 0.001) in the enzyme levels compared to the DOX-induced group (Figure 4B). 

In the estimation of nuclear factor-κB (NF-κB), analysis of the data indicated that DOX-induction significantly (*p* < 0.05) increased the levels of NF-κB in brain homogenate compared to the control group. Both tested doses of LEVE (100 and 200 mg/kg, i.p.), when administered to DOX-induced animals, significantly (*p* < 0.01) diminished the NF-κB levels compared to the DOX-induced group (Figure 4C). Further, the analysis of tumor necrosis factor-alpha (TNF-α) levels in brain homogenate suggested a significant elevation (*p* < 0.01) in DOX-induced rats compared to the control animals. When LEVE was tested at 100 and 200 mg/kg via oral administration, only the higher dose of the drug (200 mg/kg, p.o.) significantly (*p* < 0.01) reduced the elevated TNF-α levels in DOX-induced rats (Figure 4D).

### 2.5. Molecular Modelling

**Figure 5:**
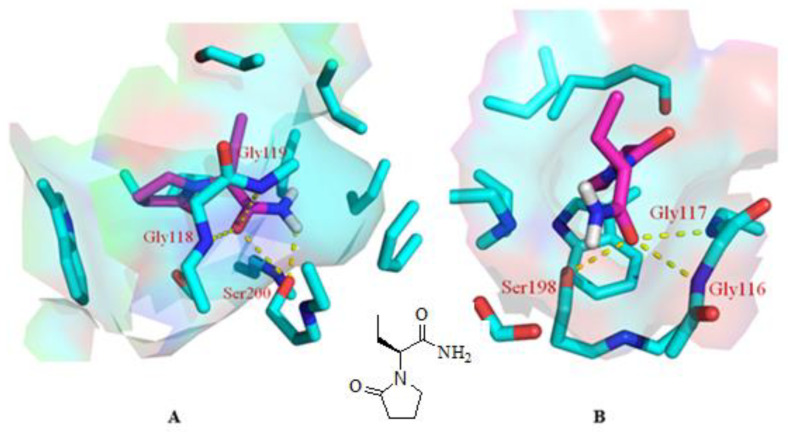
Binding mode of LEVE (pink) in the active site of AChE [(**A**), PDB code: 1DX6], BuChE [(**B**), PDB code: 4TPK] along with the 2D structure of LEVE. The yellow colored dashed line represents the hydrogen bond interactions.

As mentioned above, our experiments revealed a significant elevation in ACh levels and, hence, improvement in memory impairment through improvement in cholinergic transmission. The quest for the mechanism of action for the abovementioned effect started with inverse docking using PharmMapper. It is pertinent to mention that PharmMapper is a web-based server that is employed for finding potential targets for a molecule. The web-based query using PharmMapper indicated AChE as the potential molecular target for LEVE (Figure 5). In addition to AChE, the cholinesterase inhibition potential was also evaluated for LEVE using butyrylcholinesterase (BuChE). Hence, molecular docking was performed using the crystal structures 1DX6 and 4TPK. The docking methodology was validated by redocking the cocrystallized ligand and obtaining the binding mode as present in the crystal structure. The molecular docking of LEVE against AChE and BuChE displayed a binding score of ~ −6.00 kcal/mol for the top three binding modes. The docking score of the cocrystallized ligand was found to be −10.1 and −8.4 kcal/mol for AChE and BuChE. For the discussion, the second best binding mode was considered as it displayed interactions with key residues involved in the hydrolysis of ACh. The binding mode analysis of LEVE with AChE revealed a hydrogen bond interaction with residues Gly118, Gly119, and Ser200 (Figure 5A). The hydrophobic interactions include an alkyl-pi interaction with Trp87, Tyr124, Phe293, and Phe334. Similarly, the binding mode analysis of LEVE with BuChE reveals hydrogen bonds with Gly116, Gly117, and Ser 198 (Figure 5B). The hydrophobic interaction includes the residues Trp 231, Leu 286, Phe 329, and Phe 398.

## 3. Discussion

Chemotherapy-related cognitive deficits have become a quality-of-life issue for cancer survivors, as many suffer from memory impairment, declining executive function, loss of attention, and psychomotor speed issues during or shortly after the treatment [9]. This chemobrain is thought to be caused by oxidative stress, inflammatory processes, mitochondrial dysfunction, and glial cell interactions, among other things. Moreover, oxidative insults and peripheral TNF-α generation, in particular, are thought to be important contributors to doxorubicin-induced chemobrain [16]. In the present study, the doxorubicin (DOX) treated experimental rat model was used to explore the effect of levetiracetam (LEVE) on learning and memory behaviors. In addition, the effect of the drug on the recovery of cholinergic neuronal functions, and protection of neuronal inflammation was studied using the same model.

The results of this study showed that four weekly doses of DOX (2 mg/kg, i.p.) enhanced the latency transfer (TL) period in the elevated plus-maze (EPM) model and decreased the number of entries as well as the amount of time spent in the Y-maze arms. These two experimental models in the earlier studies were used to evaluate the spatial memory parameters [17,18,19]. The findings of these studies suggested that DOX-induction in experimental rats impaired the spatial memory parameters at the tested doses and are in accordance with the results reported in the literature [20].

DOX being a popular cancer chemotherapeutic agent is known to cause cognitive impairment in patients. The severity of the complications is reported to depend on the dose and duration of chemotherapy and can be found in patients for several weeks or months even after stopping the medications [4,5,21]. Neuroinflammation due to the hyperactivation of cytokines has been speculated to be the cause of cognitive defects. Both direct as well as indirect dysregulation in cytokine function have been linked to these defects [6,7]. In direct action, DOX-induced cytotoxicity of neuronal cells is implicated in inflammatory responses. Indirect actions that have been suggested include the activation of several pathways that occur in various parts of the body resulting in the production of inflammatory mediators [8]. The over-production and dysregulation of inflammatory mediators are reported to take place due to the action of DOX on both cancerous as well on normal cells [22].

Long-term administration of DOX is also reported to affect the microbiota of the host, which in turn disturbs the gut-brain axis leading to the activation of microglia, monocytes, and hyper-production of pro-inflammatory cytokines [5,6]. Although the entry of DOX into the brain cells is restricted by the blood-brain barrier (BBB) due to dysregulation in production and function, cytokines not only enter the brain cells to cause neuroinflammation but also damage the functional integrity of the BBB to allow the DOX entry and to induce direct action on neuronal cells [20]. The hyperactivation of neuroinflammatory mediators is evident in the present study, where the levels of COX-2, PGE2, NF-κB, and TNF-α were found to be significantly elevated compared to the control animals.

The neuroinflammation and cytotoxicity induced by chemotherapeutic agents have been associated with neurodegeneration and are known to cause alteration in the levels of acetylcholinesterase (AChE) [23]. This enzyme plays a vital role in maintaining the levels of acetylcholine (ACh) in brain cells, essential for neuronal excitation and memory function. Diminished levels of AChE are reported to cause memory impairment and interfere with the cognitive activities of patients [24]. A significant elevation in AChE levels after DOX administration suggested that defective cholinergic activity due to neuroinflammation might be responsible for cognitive impairment in the experimental animals.

LEVE is approved as an add-on drug for the treatment of partial epileptic seizures. The drug was reported to be effective in treating anxiety, neuropathic pain, absence seizures, migraine, and bipolar disorders [11]. LEVE is structurally related to the ‘nootropic drug’ piracetam [12]. In the present study, LEVE administration enhanced the spatial memory parameters of EPM and Y-maze in DOX-treated animals. The drug at both tested doses (100 and 200 mg/kg, p.o.) significantly reduced the TL values when compared with the DOX-treated group. Administration of LEVE at 200 mg/kg was also found to reverse the changes induced by DOX in terms of the number of entries in arms and time spent in the Y-maze. In earlier studies, LEVE was found to improve visual short-term memory, working memory, motor function, psychomotor speed, and concentration [13]. The present study observations support these findings, suggesting that LEVE treatment improved spatial memory and reduced the cognitive defects induced by DOX.

The present study also indicated that LEVE at the tested doses significantly reduced the COX-2, PGE2, NF-κB, and TNF-α levels in the brain homogenate of animals induced by DOX. COX-2 is a type of inducible cyclooxygenase that catalyzes the conversion of arachidonic acid to prostaglandins [25]. PGE2 plays a vital role in the inflammatory process since it causes direct vasodilation and also activates several other mediators of inflammation [26]. NF-κB is a family of highly conserved transcriptional factors that regulate several biological functions including inflammation [27]. On the other hand, TNF-α is a pro-inflammatory cytokine and causes vasodilation, edema formation, and adhesion of leucocytes to the epithelium [28]. Besides, the administration of LEVE was found to be effective in diminishing the elevated AChE levels induced by DOX. The observations suggested that LEVE at the tested doses might possess the potential to decrease the production of neuroinflammatory mediators induced by DOX. The reduction in the levels of inflammatory cytokines due to the administration of LEVE can be explained in light of several reported studies which document the effect of ACh levels on macrophage-mediated inflammatory pathways. It is very well reported that an increase in the levels of ACh results in the stimulus of α7 nicotinic ACh receptors (α7nAChR) located over macrophages or any other immune cells causing the influx of calcium ions into the macrophages. Increased levels of calcium ions activate the NF-κB pathway causing a reduction in the production of proinflammatory cytokines including TNF-α and IL-6. This is further evidenced by reports documenting anti-inflammation as an additional effect of cholinesterase inhibitors [29,30].

The inverse docking studies using PharmMapper resulted in the identification of AChE as the potential target for the drug molecule under consideration. Besides, the binding potential of LEVE was also explored against BuChE. Both the cholinesterases are present in the brain and can hydrolyze ACh. Inhibition of these cholinesterases increases the levels of Ach, thus improving cholinergic transmission. The catalytic machinery of the abovementioned two enzymes principally consists of the two most important structural features, i.e., the catalytic triad and oxyanion hole [31]. The catalytic triad in the cholinesterase is conserved and made up of Ser, His, and Glu. Similarly, the oxyanion hole is also conserved and consists of two glycines and an alanine residue [32]. Our molecular docking studies showed that the carbonyl group of LEVE interacts with the Ser200 of AChE and Ser198 of BuChE through a hydrogen bond. It is worth mentioning that the carbonyl oxygen of ACh makes a similar hydrogen bond interaction with the Ser of catalytic triad during its hydrolysis as observed in the molecular docking studies [32]. Besides, it is also to be noted that the serine of the catalytic triad facilitates the hydrolysis of ACh by the transfer of a proton from its side chain hydroxyl group to His of the catalytic triad. Our molecular docking studies also displayed a hydrogen bond interaction between the amino group of LEVE and the side chain hydroxyl group of Ser involved in the catalytic triad. LEVE also displayed a hydrogen bond interaction with Gly118, Gly 119, and Gly116, Gly117 in AChE and BuChE, respectively [33]. The Gly residues of the oxyanion hole stabilize the ACh-AChE reactant complex during the catalysis through two respective hydrogen bonds. Thus, it can be safely envisaged that the LEVE disrupts the catalytic mechanism of the cholinesterases by interacting with the key residues involved in the degradation of ACh. Considering the reported literature discussed above and the findings of the present study, it can be suggested that LEVE improved the cognitive-related parameters in DOX-induced animals and the action could be related to an enhancement in ACh levels due to the inhibition of AChE. However, more studies are suggested to support the nootropic potential of LEVE against cancer chemotherapy-induced cognitive impairment.

## 4. Materials and Methods

### 4.1. Experimental Animals

In the present experiment, a total number of twenty-eight adult male Sprague Dawley rats aged three months (150–200 g body weight) were used. The rats were divided into four groups and each group was limited to seven animals (n = 7). The sample size was decided based on our previously published reports and the methods reported by Arifin and Zahiruddin (2017) [33,34,35]. The animal experiments followed the guidelines of the College Pharmacy Research Center (Ethical Approval ID 2020-CP-14) and the Deanship of Scientific Research of Qassim University under grant number 10223-pharmacy-2020-1-3-I. The animals were stored in the animal house facility of the College of Pharmacy as per the standard guidelines. The animals were maintained with a 12 h light-dark cycle for seven days of acclimatization and thirty days of experimental duration. A maximum of four rats were housed in a standard polypropylene cage and maintained in a pathogens-free environment. The room temperature was maintained at 22 ± 2 °C. All the rats were allowed free access to water and standard rodent pellet food (First Milling Company, Jeddah, Saudi Arabia).

### 4.2. Experimental Groups

**Figure 6:**
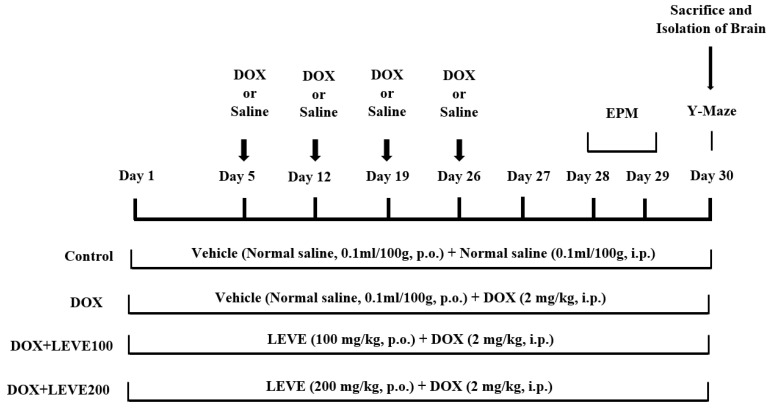
Timeline administration of drug, behavioral assessments, and isolation of brain samples. The selected groups of rats were administered the vehicle or levetiracetam (LEVE) for thirty days by oral administration. Except for the control, the other groups were injected with four doses of DOX (2 mg/kg, i.p.) for inducing neurotoxicity (days 5, 12, 19, and 26). Regarding elevated plus-maze (EPM) assessments, the training sessions were conducted on day 28, and memory assessments were analyzed on day 29. Whereas, both sessions of the Y-maze test were conducted on day 30. At the end of the memory tests, on day 30, all of the animals were sacrificed, and brain tissues were collected for ELISA tests.

A total number of twenty-eight rats were divided into four groups (n = 7). The control group of animals were treated with normal saline (0.1 mL/100 g, p.o.) for 30 continuous days and also received four doses of intraperitoneal (i.p.) injection of normal saline (0.1 mL/100 g, i.p.) on the 5th, 12th, 19th, and 26th days of the drug treatment schedule. The second group was DOX-induced, administered with normal saline (0.1 mL/100 mg, p.o.) for 30 continuous days, and also received doxorubicin [(DOX), ADRIN^®^, Fresenius Kabi Oncology Ltd., Pune, India] 2 mg/kg by intraperitoneal (i.p.) injection once a week for four weeks [36]. The remaining two groups were the drug treatment groups; these rats were treated with 100 or 200 mg/kg of levetiracetam [(LEVE), Toronto Research Chemicals, Canada] for 30 days, and neurotoxicity was induced with concomitant administration of doxorubicin (2 mg/kg/week, i.p.), similarly to DOX-induced. The doses of LEVE were selected based on the previous reports [37]. The DOX and LEVE were dissolved in a sterile isotonic saline solution for treatment. An elevated plus-maze (on the 28th and 29th days of drug treatment) and a Y-maze test (on the 30th day) were used to examine the spatial memory of the animals. All animals were sacrificed after the maze testing (on the 30th day), and the entire brain was obtained for various biochemical assessments (Figure 6).

### 4.3. Assessment of Spatial Memory

#### 4.3.1. Elevated Plus-Maze (EPM)

EPM is a behavioral paradigm for measuring rat memory that is extensively employed. Two of its arms are closed, while the other two are open. The maze is raised 50 cm high from the ground during the experiment since rodents dislike staying in the open and elevated arm and prefer to explore and stay in the enclosed arm. Transfer latency (TL), the time taken by rats to enter the enclosed arm from any one of the open arms, was estimated on the training day (28th day of drug treatment) as well as on the experiment day (29th day of drug treatment). If the rat does not move into a closed arm within 90 s on training day, the animal is guided to one of the closed arms and allowed to stay in the maze for two minutes. After 24 h, memory retention was assessed [17,35,38].

#### 4.3.2. Y-Maze Test

The Y-maze test was used to assess the rats’ ability to recognize the novel arm and their proclivity to explore new environments. The wooden Y-maze has three arms and measures 50 × 10 × 30 cm in length, width, and height. Each arm is at a 120-degree angle from the others. One arm (considered a novel arm) was closed during the training session (30th day of treatment), and the animals were free to explore for 5 min in the other two arms. During the training session, the number of entries into two arms was recorded. The entry of the animal into an arm was considered if it entered 85% of its body. At an interval of 4 h, the novel arm was opened, and the rats were given 5 min to explore again. The total number of entries into all three arms, as well as the time spent in the new arm, were recorded during the test session. The number of entries into the novel as well as the known arms, the time spent in the novel arm, and the total number of entries in all three arms were recorded during the test session. The total time spent in the novel arm divided by the total time spent in all arms during the test session was referred to as the proportion of time spent in the novel arm [17,19,34].

### 4.4. Enzyme-Linked Immunosorbent (ELISA) Assays

#### 4.4.1. Brain Samples Collection

All animals were sacrificed by cervical decapitation under light ether anesthesia on day 30, after the last dose of the drug administration and the Y-maze examination. The entire fresh brain was immediately removed from the skull and homogenized with phosphate buffer (pH 7.6, 4 °C). After centrifugation for 10 min at 4000 rpm, the cloudy supernatant aliquot was collected. The cholinergic and neuroinflammatory biomarkers were determined using the homogenate obtained, as described in the following sections.

#### 4.4.2. Acetylcholinesterase (AChE)

An ELISA kit from MyBioSource (Lot # L210111436, MyBioSource Inc., San Diego, CA, USA) was used to estimate AChE levels in brain homogenate. The assay is based on the interaction of the antigen (AChE) with an antibody specific to AChE, which results in a color shift that can be detected spectrophotometrically at 450 nm [39]. In brief, the test method includes the addition of samples, various concentrations of standard, and a blank (PBS) into the pre-determined wells of the kit’s plate. Then, the successive addition of different reagent solutions was performed, such as horseradish peroxidizes, TMB (tetramethylbenzidine), and Avidin as per the method described in the kit manual. Washing was performed to remove excess reagents, and incubation was performed to help the reagents react together. Finally, the AChE concentration was estimated by measuring the optical density of the color generated in the solution and comparing it to the reference curve.

#### 4.4.3. Prostaglandin E2 (PGE2)

The assay was carried out with the rat prostaglandin E2 ELISA kit purchased from MyBioSource (Lot # R1800G014, MyBioSource Inc., San Diego, CA, USA). The principle of the procedure is based on the color change in the solution caused by the reaction of PGE2 (antigen) with a biotinylated detection antibody in the presence of horseradish peroxidize-streptavidin (SABC). This was measured spectrophotometrically at 450 nm and compared with the standard to determine the concentration [26]. In brief, the procedure includes the addition of different concentrations of standard, sample, and blank into the wells. Several reagents were successively added as per the method described in the kit manual, such as biotin-labeled antibody solution, SABC, TMB (tetramethylbenzidine), and stop solution. Every addition was followed by incubation at different time intervals at 37 °C and washing with wash buffer. Finally, the optical density of the color developed in the solution was recorded at 450 nm. The content of the PGE2 in the samples was determined by using the standard curve plotted against different concentrations.

#### 4.4.4. Cyclooxygenase-2 (COX-2)

An ELISA kit from MyBioSource (Lot # 20202019, MyBioSource Inc., San Diego, CA, USA) was used to assess the quantity of COX-2 in brain homogenate. The estimating principle is based on the reaction between the antigen (COX-2) and the antibody, which results in the production of color at 450 nm, which can be detected spectrophotometrically [25]. Briefly, the samples, different concentrations of standard, and blank (PBS) were added into the rat COX-2 antibody pre-coated wells. For detecting the optical density, streptavidin-horseradish peroxidize was used to augment the color development. Incubation of the plate after every addition of reagents was performed as per the kit’s manual to assist in the reaction between the different reagents. Using the standard curve, the intensity of the color developed was utilized to determine the amount of COX-2 in the sample. 

#### 4.4.5. Nuclear Factor-Kappa Beta (NF-κB)

The estimation was performed using the ELISA kit purchased from MyBioSource (Lot # R1186G014, MyBioSource Inc., San Diego, CA, USA). The estimating principle is based on a sandwich enzyme-linked immunosorbent assay in which NF-κB in the samples was used as an antigen and its reaction with a pre-coated antibody resulted in a color change in the presence of numerous detecting reagents. The development of color was measured spectrophotometrically at 450 nm [27]. The brief procedure of assay includes the addition of samples, and different concentrations of standard and blank (PBS) into the wells of a microtiter plate. Different reagents, such as a biotin-labeled antibody, horseradish peroxidize-streptavidin, and TMB (tetramethylbenzidine) were added successively as per the method mentioned in the user manual. Incubation was performed at different time intervals to augment the reaction, and washing was performed to remove the excess reagent. Finally, an acidic solution was added to terminate the reaction, and the color formed in the solution was spectrophotometrically quantified at 450 nm. The standard curve created from different concentrations was utilized to determine the amount of NF-κB in the samples.

#### 4.4.6. Tumor Necrosis Factor-Alpha (TNF-α)

An ELISA kit from MyBioSource (Lot # CK6E39B, MyBioSource Inc., San Diego, CA, USA) was employed to estimate the TNF-α content. The present estimation is based on the antigen (TNF-α) and antibody response, which is evaluated spectrophotometrically at 450 nm as a color change [28]. In brief, the procedure includes the addition of samples, different concentrations of standard, and blank (PBS) to the pre-determined wells in the plate. Various reagents, such as a biotinylated antibody, horseradish peroxidize-conjugated streptavidin, and TMB (tetramethylbenzidine) were added successively as per the methods described in the kit manual. Incubation to augment the reaction and washing to remove the excess reagents were performed. Finally, the color developed in the solution was measured and the optical density was recorded at 450 nm. The intensity of the color was compared with the standard to determine the concentration of TNF-α in the samples.

### 4.5. Statistical Analysis

Results of all in vivo studies using rats were stated as mean ± SEM (standard error). The collected data was evaluated using one-way ANOVA and the significant level between the groups was analyzed with the Tukey–Kramer post hoc test. A probability value of 0.05 was considered significant.

### 4.6. Molecular Modelling

Molecular docking was performed using AutoDock Vina integrated with PyRx [40]. The crystallized proteins with pdb code 1DX6 (AChE) and 4TPK (hBuChE) were downloaded from the protein data bank. The input files for molecular docking were prepared using AutoDock tools bundled with MGL tools (version 1.5.6) [41]. Protein was prepared by removing cocrystallized ligands, and water molecules. The proteins were corrected for missing atoms and for spatially close residues. The polar hydrogen atoms and charges were assigned. The 3D structure of LEVE was retrieved from the PubChem database as a single file in 3D-spatial data file (SDF) format. The structure of LEVE was imported in the open babel interface of PyRx and minimized using a universal force field and saved in PDB format. The gasteiger charges and polar hydrogens were added, and LEVE was set up for the rotatable bond. The prepared protein and ligand files were then converted into PDB format, which serves as an input file for AutoDock Vina for molecular docking. The grids were generated with the autogrid program distributed with AutoDock, (version 1.5.6). The binding site of the ligand was chosen as the active grid centre. The dimensions were chosen to include all atoms of the ligand. The size of the grid box includes 25 Å distance in X, Y, and Z directions. The docking methodology was validated by reproducing the binding mode of the cocrystallized ligand. The molecular docking studies were followed in reference to previously reported studies [42,43]. The inverse docking studies were performed using a web-based server PharmMapper [44]. 

## 5. Conclusions

In the present study, DOX-treated rats showed cognitive impairment characteristics in an elevated plus maze and Y-maze. LEVE was tested in two doses (100 and 200 mg/kg, p.o.) against the DOX-induced memory defects. The higher dose of LEVE (200 mg/kg) was found to be more effective in reducing the cognitive defects induced by DOX and the treatment also decreased acetylcholinesterase and the levels of neuroinflammatory mediators such as COX-2, PGE2, NF-κB, and TNF-α in brain homogenate. The observations suggested the nootropic-like action of LEVE and can be related to enhanced cholinergic activity and diminished neuroinflammatory mediator levels. The molecular modelling studies suggested AChE as the potential target for LEVE, which interferes with the catalytic cycle by interacting with the residues of the catalytic triad and the residue responsible for the stabilization of the ACh and AChE reactant complex. However, more studies are needed to establish the actual potential of LEVE against cancer chemotherapy-induced cognitive impairment.

## Data Availability

The data presented in this study are available from the corresponding author upon reasonable request.

## Sample Availability

Samples of the compounds are not available from the authors.
